# Supplementary material for: Isookanin Inhibits PGE2-Mediated Angiogenesis by Inducing Cell Arrest through Inhibiting the Phosphorylation of ERK1/2 and CREB in HMEC-1 Cells
Source: Int J Mol Sci. 2021 Jun 16;22(12):6466. doi: 10.3390/ijms22126466 (PMC8234715; doi:10.3390/ijms22126466)
Supplement: Supplementary file 1 [file ijms-22-06466-s001.zip › ijms-1239651-supplementary.pdf]

Supplementary material

Methods

PGE$_2$ receptor, EP4, expression was determined by immunocytochemistry. Aliquots of 1.2 × 10^5 cells were seeded into 24-well plates and cultured overnight. And then the cells were treated in the presence or absence of isookanin (1, 5 and 10 μg/mL) for 1 h before stimulation with PGE$_2$ for 24 h. After incubation, the cells were fixed in 4% paraformaldehyde, washed with PBS, permeabilized with 0.2% Triton X-100, and incubated with a mouse monoclonal antibody to EP4 (1: 100, 5% BSA in PBS) for 2 h, followed by fluorescence staining with fluorescein isothiocyanate Alexa Fluor®488 (1: 200) (Santa Cruz Biotechnology, Santa Cruz, CA, USA) for 1 h. After three washes with PBS, the cells were incubated with 1 μg/mL Hoechst 33342 (Invitrogen, Carlsbad, Calif, USA) for 10 min. The cells were then examined using the EVOS fluorescent microscope (Advanced Microscopy Group, Bothell, WA, USA.).

Results

Effect of isookanin on EP4 expression in PGE$_2$-induced HMEC-1 cells

PGE$_2$ binds to and activates 4 cognate receptors named EP1, EP2, EP3, and EP4. Among them, EP4 has been reported to play an important role in angiogenesis. To investigate whetherisookanin affects PGE$_2$-induced EP signaling, the cell surface expression of EP4 on HMEC-1 cells was assessed by immunohistochemistry. The results revealed that PGE$_2$-only treated endothelial cells showed increased EP4 expression, while cells pretreated with isookanin before PGE$_2$ stimulation showed a significant and dose-dependent decrease in EP4 expression.

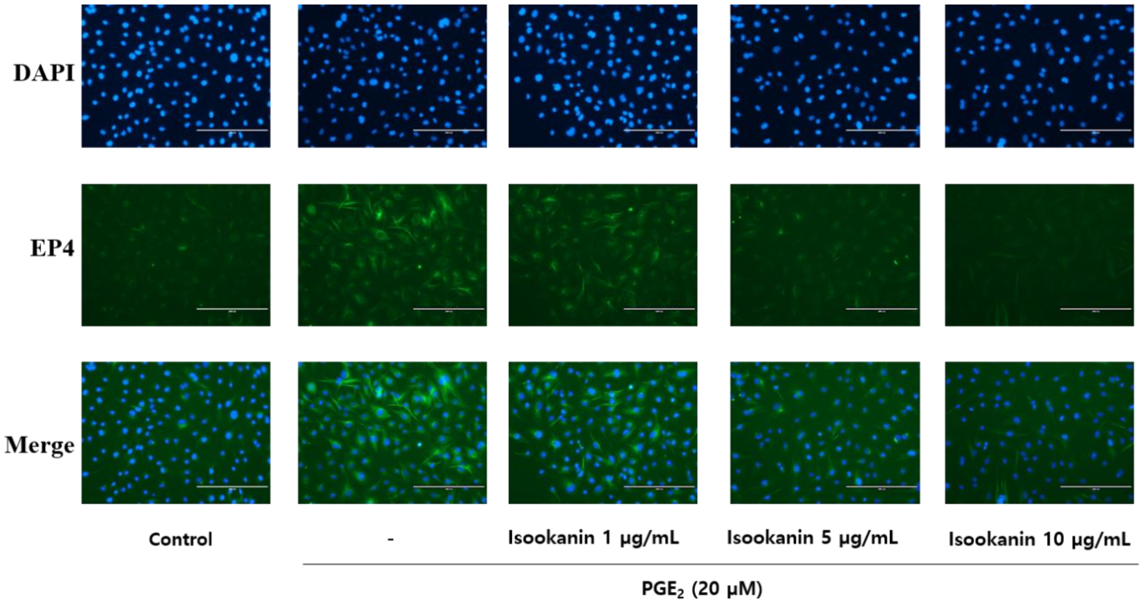

Figure S1. Effects of isookanin on EP4 expression in PGE$_2$-induced HMEC-1 cells. Cells were pretreated with the indicated concentrations of isookanin for 1 h, before stimulation with PGE$_2$ (20 μM) for 24 h. Then incubated with a mouse monoclonal antibody to EP4 (1: 100, 5% BSA in PBS) for 2 h, followed by fluorescence staining with fluorescein isothiocyanate Alexa Fluor®488 (1: 200) for 1 h. After three washes with PBS, the cells were incubated with 1 μg/mL Hoechst 33342 for 10 min. The cells were then examined using the EVOS fluorescent microscope, scale bars is 1000 μm.
